# Effect of Antithrombin Leakage From Hemodialysis Therapy on Antithrombin Hemodynamics

**DOI:** 10.7759/cureus.75117

**Published:** 2024-12-04

**Authors:** Yoshinari Fujii, Satomi Nagaya, Atsunobu Seo, Yuji Kanazawa, Taisei Oba, Eriko Morishita

**Affiliations:** 1 Faculty of Health and Medical Sciences, Hokuriku University, Kanazawa, JPN; 2 Clinical Laboratory Science, Graduate School of Medical Science, Kanazawa University, Kanazawa, JPN; 3 Hemodialysis, Itaya Clinic, Kaga, JPN; 4 Faculty of Health and Medical Sciences, Hokuriku university, Kanazawa, JPN

**Keywords:** antithrombin, hemodiafiltration, hemodialysis, heparin, leakage

## Abstract

Introduction

Hemodialysis (HD) therapy is a crucial treatment for patients with renal failure but can impact the hemodynamics of antithrombin (AT), a protein essential for regulating hemostasis and preventing thrombosis. Reduced AT activity can lead to thrombus formation at unusual sites and increase the risk of recurrent venous thromboembolism. The loss of AT during HD or hemodiafiltration (HDF) through leakage or adsorption onto dialysis membranes has not been fully investigated, and its effects on AT hemodynamics remain unclear. We aimed to elucidate the mechanisms underlying AT activity reduction due to dialysis, with the goal of developing dialysis protocols that preserve AT activity and reduce the risk of vascular access-related thrombosis.

Methods

AT activity and antigen levels were measured before and after dialysis therapy in 24 patients undergoing maintenance dialysis at Itaya Clinic (HD, 12; HDF, 12). AT antigen levels in dialysis effluent were also measured to analyze the effects of dialysis on AT hemodynamics. Additionally, immunofluorescence staining of dialysis membranes was used to semi-quantitatively assess the amount of AT adsorbed onto the membrane.

Results

AT activity and antigen levels in patients undergoing HD were significantly lower than those in healthy participants but increased following dialysis. A negative correlation was found between dialysis vintage (history of heparin use) and predialysis AT activity. AT leakage and adsorption were significantly greater with HDF than with HD. However, no correlation was observed between AT leakage and activity or antigen levels before and after dialysis.

Conclusions

AT activity and antigen levels were decreased in patients on HD, with long-term heparin use suggested as a contributing factor. Additionally, AT leakage was observed during HDF therapy, indicating that dialysis-related AT leakage may contribute to decreased AT activity and antigen levels. Therefore, regular measurement of AT activity is recommended for patients with HD. If AT activity decreases, treatment adjustments, such as switching to HD therapies that minimize AT leakage and adsorption, should be considered.

## Introduction

In blood purification therapy, the target molecular weight for substance removal has evolved over time. In 1985, the identification of β2-microglobulin (β2-MG), with a molecular weight of 11,800, as the cause of dialysis-related amyloidosis [1], drew attention to β2-MG as a clinical target for removal. Protein-bound toxins, which bind to plasma proteins, such as human serum albumin, are associated with mortality, cardiovascular morbidity, and pruritus [2], and the target for removal has been expanded to the molecular weight range of albumin (66,000). In 2021, Rosner et al. proposed a uremic toxin classification that divides toxins into six fractions: small protein-bound molecules (<0.5 kDa), small molecules (<0.5 kDa), small-middle molecules (0.5-15 kDa), medium-middle molecules (15-25 kDa), large-middle molecules (25-58 kDa), and large molecules (>58-170 kDa), and also proposed a suitable removal therapy for each type of uremic toxin [3]. Hemodiafiltration (HDF) is the predominant form of dialysis in Japan [4]. Regarding the efficacy of HDF therapy, numerous studies have reported favorable data on dialysis-related hypotension [5, 6], restless leg syndrome [7], and patient survival [8-10]. However, depending on the hemodiafilter used, the convection volume, and other conditions, there is a possibility that substances with a molecular weight similar to albumin may also be inadvertently removed [11].

Antithrombin (AT), a serine protease inhibitor with a molecular weight of approximately 58,000, is synthesized in the liver and serves as a natural anticoagulant critical for hemostasis and thrombosis [12]. It is present in normal plasma at a concentration of 15-31 mg/dL and primarily regulates the activities of thrombin and activated factor X. This anticoagulant activity is enhanced approximately 1,000-fold by reaction with heparan sulfate on vascular endothelial cells. AT deficiency can result in venous thromboembolism [13] and thrombosis at unusual sites such as the portal vein [14] or cerebral venous sinuses [15], leading to recurrent thromboembolism. Decreased AT activity and antigen levels have been reported in patients undergoing hemodialysis (HD) [16] and decreased AT activity has been linked to a shorter primary patency of arteriovenous fistulas [17]. The enhanced clearance potential of HDF may reduce AT antigen levels due to AT leakage from plasma to the dialysis side through the dialysis membrane and membrane adsorption, yet limited research has investigated AT hemodynamics during dialysis.

In 2020, the International Society on Thrombosis and Haemostasis, Inc. reported that the causes of acquired AT deficiency include liver dysfunction, excessive consumption, protein leakage into the urine associated with nephrotic syndrome, long-term heparin use, malnutrition, and dilutional effects [18]. In blood purification, factors such as long-term heparin use, consumption, and dilutional effects are relevant. Crupp et al. attributed the high prevalence of anticoagulant factor deficiencies in patients undergoing HD to increased consumption of anticoagulant factors (AT, protein C, and protein S) resulting from enhanced coagulation activity associated with chronic kidney disease and dialysis therapy [19]. However, AT leakage and adsorption on dialysis membranes remain underexplored. Therefore, to clarify the effect of dialysis on AT hemodynamics, we measured AT activity and antigen levels before and after dialysis, as well as AT antigen levels in dialysis effluent. We also performed a semi-quantitative analysis of AT proteins adsorbed onto the dialysis membrane. In this study, we aimed to elucidate the mechanisms underlying AT activity reduction due to dialysis, with the goal of developing dialysis protocols that preserve AT activity and reduce the risk of vascular access (VA) related thrombosis.

## Materials and methods

Study participants

The study included 90 patients undergoing HD at Itaya Clinic. Within this group, 24 patients were evaluated, excluding 66 patients who did not give consent to participate or instances where fibrin was deposited during the thawing of frozen plasma. Additionally, 32 healthy participants (19 men and 13 women), aged between 30 and 80 years and employed at Hokuriku University, served as controls. Approval for the study was granted by the Hokuriku University Ethics Committee for Research Involving Human Subjects (approval numbers 2022-3), and written informed consent was obtained from all participants.

Data collection

Patient blood samples were collected during routine blood draws on the first day of dialysis each week from April to June 2023. Samples from healthy participants were obtained during a staff health check in May 2023. Samples were drawn using coagulant-containing tubes (Sekisui Medical Co., Ltd., Tokyo, Japan), EDTA-2K-containing tubes (Sekisui Medical Co., Ltd.), and 3.2% citrate-containing tubes (Sekisui Medical Co., Ltd.). Patient samples were taken from the blood access needle immediately before dialysis and directly from the blood circuit after dialysis. Healthy participant samples were drawn from the median vein using a 22G needle (Terumo, Tokyo, Japan). All blood samples were immediately centrifuged at 1500 × g for 10 minutes at room temperature, after which the serum and plasma were retrieved and stored at -80°C until analysis. Dialysate effluent was continuously collected at 100 mL/h during dialysis treatment and stored at -80°C until measurement.

Blood samples were analyzed using the following reagents: Test Team S ATIII for AT activity, NS Auto ATIII (Type B) for AT antigen, LZ Test 'Eiken' α1-M for α1-microglobulin (α1-MG), LZ Test 'Eiken' β2-M for β2-MG, Nanopia SF for soluble fibrin (SF), Nanopia D-dimer for D-dimer, and HISCL™ TAT Reagent for thrombin-antithrombin complex (TAT). Hematocrit was measured by centrifugation. Dialysate samples were analyzed using the N-assay TIA Micro Alb Nittobo E-Type for albumin levels, and AT antigen levels were measured via enzyme-linked immunosorbent assay using paired antibodies from the Antithrombin-ATIII Paired Antibody Set (Affinity Biologicals Inc., Ancaster, Canada).

Patient data and dialysis information such as age, sex, dialysis vintage, cause of kidney disease, complications, diabetes mellitus status, anticoagulant use, dialysis mode, dry weight (DW), the quantity of blood flow, and type of dialyzer or hemodiafilter, were collected. Routine blood samples were also collected to determine albumin, creatinine, and urea nitrogen levels.

Calculation of reduction rates and leakages

The reduction rates (RR), including hematocrit correction for AT antigen, a1-MG, b2-MG, and albumin, were calculated as follows:

$RR(\%)=\left\{ 1-\left[ HCTpre\times \left( 1-HCTpost/100 \right)\times Cpost/HCTpost\times \left( 1-HCTpre/100 \right)\times Cpre \right] \right\}\times 100$

Cpre and Cpost indicate the solute concentration of AT antigen, a1-MG, b2-MG, and albumin before and after dialysis, respectively, while HCTpre and HCTpost indicate the hematocrit values before and after dialysis, respectively.

The amount of AT antigen and albumin leakage was calculated using the following formula:

$Leakage\ amount(\%)=\left[ concentration \ of \ solutes\ in\ the\ dialysate (\text{mg/dL})\right]\times \left[ dialysate\ flow\ rate(\text{L/min}) \right]\times \left[ dialysis\ time(minutes) \right]$

Immunofluorescence of dialysis membranes

**Figure 1:**
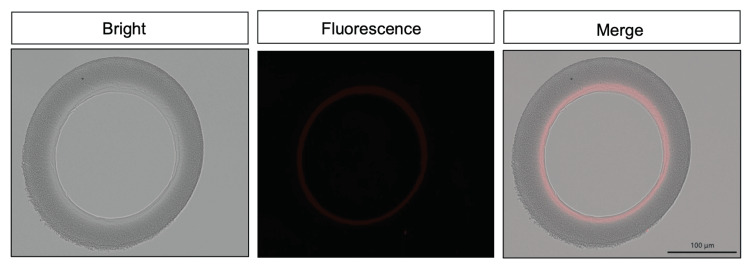
Immunofluorescence stained images of hemodialysis membranes. Hemodialysis membranes were sectioned into frozen slices and immunofluorescently stained with anti-thrombin antibodies. The membranes were imaged and the area of the hollow fibers was determined using bright-field imaging. The sum of the fluorescence intensities within the determined area was considered as the amount of adsorbed antithrombin.

Immunofluorescence was performed to semi-quantify AT proteins adsorbed on the dialysis membrane. We collected the dialyzer and hemodiafilter immediately after the dialysis sessions. Each dialyzer and hemodiafilter was mechanically broken down, and the hollow fiber membranes were collected separately from the blood inlet and outlet sides. The collected hollow fiber membranes were stored in a 10% neutral-buffered formalin solution. Transverse sections (10 µm thick) were cut from the hollow fiber membranes using a cryostat (CM1950; Leica, Wetzlar, Germany) at -25 °C and the sections were placed in 600 μL tubes. Subsequently, the sections were washed three times with phosphate-buffered saline (PBS; pH 7.4). The sections were then incubated with Anti-Antithrombin III, Rabbit (GeneTex, California, USA) diluted 500-fold in PBS at 4 °C for 24 hours. Subsequently, the sections were incubated with Anti-rabbit lgG (H+L), F(ab')2 Fragment (Alexa Fluor® 555 Conjugate, Cell Signaling Technology, Massachusetts, USA ) diluted at 1:1000 in PBS at 25 °C for 1 hour. After washing three times with PBS, they were sealed with the water-soluble sealant Fluoromount (Diagnostic BioSystems, California, USA). The fluorescently stained sections were observed under a microscope (BZ-X800; Keyence, Osaka, Japan). First, a range of hollow fiber membranes was extracted in a bright field, and the total fluorescence intensity within the hollow fiber membrane was measured (Figure 1). Fluorescence intensities from three to five membranes were calculated and averaged. Next, AT adsorption was calculated by subtracting the control (unused hollow fibers) from the measured fluorescence intensity.

Statistical analysis

Statistical analyses were performed using IBM SPSS Statistics v.29 software (IBM, New York, USA). Group differences were assessed using the Student’s t-test for normally distributed variables, the Mann-Whitney U test for non-normally distributed variables, and the chi-squared test for categorical data. Continuous variables are presented as means ± standard deviations or as medians with interquartile ranges (IQR). Statistical significance was set at p < 0.05.

## Results

Patient characteristics and dialysis conditions

The median age of the patients on HD who participated in this study was 70 (IQR 56-75) years, with 14 men and 10 women. The median dialysis vintage was 63.0 (IQR 36.0-97.5) months (Table 1). Among the participants, 12 were on HD, and 12 were on pre-diluted online HDF. Three types of dialyzers were used: the FX series (polysulfone membrane, Fresenius Medical Care, Bad Homburg, Germany), the FB-Uβ series (cellulose triacetate membrane, Nipro Co., Osaka, Japan), and the NF-HS series (polymethyl methacrylate membrane, Toray Medical Co., Ltd., Tokyo, Japan). Additionally, three types of hemodiafilters were used: the ABH-LA series (polysulfone membrane, Asahi Kasei Medical Co.), FIX-S series (asymmetric triacetate membrane, Nipro Co.), and FIX-E series (asymmetric triacetate membrane, Nipro Co.). Patients on HDF had a significantly higher DW (p = 0.012) and longer dialysis times (p = 0.017) than those on HD. In all pre-diluted online HDF cases, the convection volume was 10 L/h.

**Table 1:** Patient information and hemostatic parameters in patients on hemodialysis and hemodiafiltration. HD: hemodialysis; HDF: hemodiafiltration; QB: quantity of blood flow; BUN: blood urea nitrogen; TAT: thrombin-antithrombin complex.

	All patients	Patients on HD	Patients on HDF	p-value for HD/HDF comparisons
	n =24	n =12	n =12	
Age (y/o)	70.0 (56.0-74.5)	68.0 (56.5-75.0)	70.5 (55.0-74.5)	0.799
Sex (male)	14	5	9	0.098
Dialysis vintage (months)	63.0 (36.0-97.5)	62.5 (35.0-107.5)	65.0 (36.0-93.5)	0.887
Cause of kidney disease				0.494
Deabetic nephropathy	10	4	6	
Glomerulonephritis	4	1	3	
Nephrosclerosis	2	1	1	
IgA nephropathy	2	1	1	
Others	4	4	0	
Unknown	2	1	1	
Dry Weight (kg)	56.3 (48.0-65.0)	49.3 (42.4-58.3)	62.5 (55.8-72.3)	0.012
Dialysis time (hours)	4.00 (3.50-4.00)	3.75 (3.00-4.00)	4.00 (4.00-4.50)	0.017
QB (mL/min)	200 (200-200)	200 (190-200)	200 (200-200)	0.089
Kt/V	1.36 ± 0.21	1.35 ± 0.26	1.37 ± 0.16	0.773
Anticoagulant				1
Unfractionated heparin	22	11	11	
Low molecular weight heparin	2	1	1	
Albumin (g/dL)	3.70 (3.50-3.85)	3.70 (3.43-3.90)	3.65 (3.50-3.80)	0.713
Creatinine (mg/dL)	10.9 ± 0.7	9.9 ± 3.0	12.0 ± 3.5	0.12
BUN (mg/dL)	64.1 ± 3.0	65.2 ± 17.4	63.0 ± 12.5	0.729
Pre antithrombin activity (%)	77.8 ± 11.4	78.1 ± 13.2	77.6 ± 10.0	0.918
Pre antithrombin antigen (mg/dL)	26.5 ± 3.7	26.9 ± 4.0	26.0 ± 3.4	0.214
Soluble fibrin (mg/mL)	51.0 ± 21.9	52.3 ± 22.8	49.8 ± 21.8	0.787
D-dimer (mg/mL)	1.5 (1.4-2.9)	1.6 (1.2-3.4)	1.5 (1.5-2.4)	0.932
TAT (ng/mL)	3.0 (2.1-6.0)	4.1 (2.3-12.7)	2.6 (1.9-5.4)	0.347

AT activity and antigen levels in patients on HD and healthy participants

The pre-dialysis (pre) AT activity and antigen levels in patients on HD were 77.8 ± 11.4% and 26.5 ± 3.7 mg/dL, respectively (Table 1). Pre-AT activity was below the reference range of reagents (80-130%) in 11 of 24 (45.8%) patients, and pre-AT antigen levels were below the reference range (23.6-33.5 mg/dL) in six of 24 (25.0%) patients. No correlation was observed between pre-AT activity or antigen levels, and patient age or sex. AT activity and antigen levels in healthy controls (53 (IQR 41-65) years, 19 men and 13 women) were 100.9 ± 9.8% and 32.8 ± 2.1 mg/dL, respectively, and AT activity and antigen levels were significantly lower in patients on HD (p < 0.001, both). SF levels were elevated in all patients, with an average of 51.0 ± 21.9 mg/mL (reference range: <7.0 mg/mL). TAT levels averaged 3.0 (2.1-6.0) ng/mL, exceeding the reference range (<4.0 ng/mL) in 10 of 24 patients. D-dimer levels, indicating the presence of thrombus, were 1.5 (1.4-2.9) mg/mL, which was above the reference range (<1.0 mg/mL) in 21 of 24 patients. However, no correlation was observed between AT activity or antigen levels and SF, TAT, or D-dimer levels.

Changes in AT activity and antigen levels before and after dialysis and reduction rates

**Figure 2:**
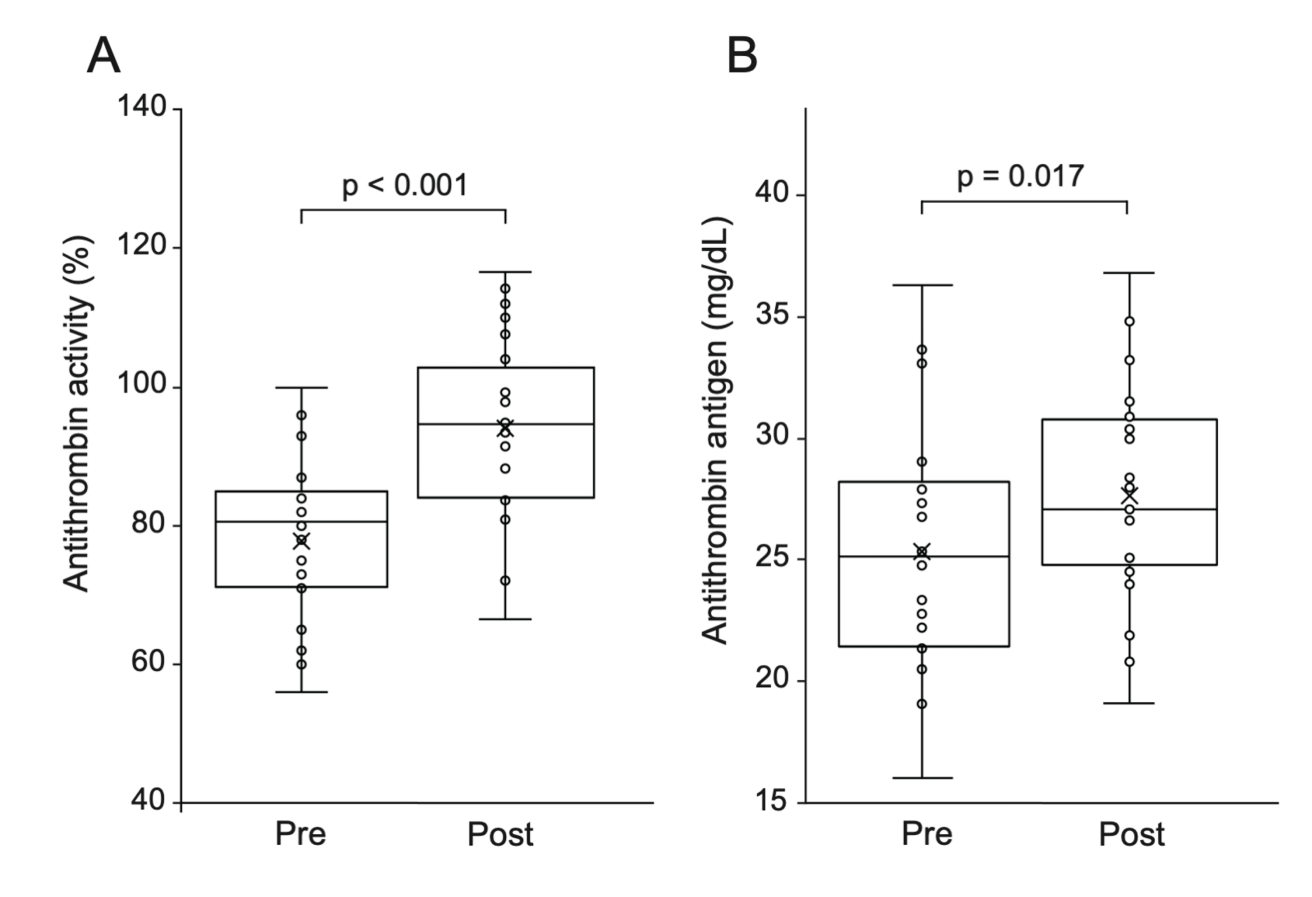
Changes in AT activity and antigen levels before and after dialysis. A: AT activity increased significantly with dialysis treatment. B: AT antigen levels increased significantly with dialysis. AT: antithrombin.

After dialysis, AT activity increased in 20 of 24 patients, and AT antigen levels increased in 17 of 24 patients, with both values significantly higher than pre-dialysis levels (Figure 2, Table 2, p <0.001, p = 0.017, respectively). Albumin levels were elevated after dialysis in all patients (p < 0.001). There was no significant difference in the AT reduction rate after hematocrit correction between HD and HDF therapies (p =0.214). Additionally, there was no correlation between the AT reduction rate and pre-AT activity or pre-AT antigen levels (p = 0.884, R = 0.032, p = 0.431, R = 0.169, respectively).

**Table 2:** Antithrombin activity, antigen levels, albumin, α1-microglobulin, and β2-microglobulin before and after dialysis and their reduction rates. HD: hemodialysis; HDF: hemodiafiltration.

	Dialysis mode	Pre dialysis therapy	Post dialysis therapy	Reduction rate (%)	p-value for pre/post comparisons	Effect size	p-value for HD/HDF reduction rate comparisons	Effect size
Antithrombin activity (%)	ALL	77.8 ± 11.4	89.0±16.9	−	< 0.001	0.67		
	HD	﻿78.1 ± 13.2	91.8 ± 17.4	−	0.001	0.79		
	HDF	﻿77.6 ± 10.0	86.2 ± 16.6	−	0.057	0.54		
Antithrombin antigen (mg/dL)	ALL	26.5 ± 3.7	27.6 ± 4.3	16.0 ± 7.6	0.017	0.47		
	HD	26.9 ± 4.0	28.0 ± 4.5	14.0 ± 8.2	0.158	0.42	0.214	0.26
	HDF	26.0 ± 3.4	27.3 ± 4.1	17.9 ± 6.6	0.063	0.53		
Albumin (g/dL)	ALL	3.7 (3.5-3.9)	4.3 (3.9-4.5)	7.6 (3.0-10.4)	< 0.001	0.86		
	HD	3.7 (3.5-3.9)	4.3 (3.9-4.5)	5.7 (0.6-11.6)	0.003	0.85	0.59	0.11
	HDF	3.7 (3.5-3.8)	4.3 (4.0-4.7)	7.7 (5.4-10.4)	0.002	0.89		
α1-microglobulin (mg/dL)	ALL	129.8 (118.4-143.4)	127.5 (96.8-163.1)	19.3 (5.5-33.5)	0.689	0.08		
	HD	134.8 (121.3-150.3)	154.0 (124.0-181.5)	5.5 (2.4-15.4)	0.01	0.75	p < 0.001	0.68
	HDF	121.6 (115.0-136.7)	105.6 (93.9-130.5)	33.5 (19.3-37.9)	0.05	-0.57		
β2-microglobulin (mg/dL)	ALL	32.9 (27.6-37.1)	12.9 (8.0-19.1)	67.0 (57.7-77.0)	< 0.001	-0.88		
	HD	35.6 (33.8-38.4)	19.1 (15.6-20.2)	57.7 (45.1-64.0)	0.002	-0.88	p < 0.001	0.85
	HDF	29.6 (25.2-32.6)	8.0 (6.9-8.7)	77.0 (74.5-80.0)	0.002	-0.88		

Effects of long-term heparin use and fluid overload on AT activity and antigen levels

**Figure 3:**
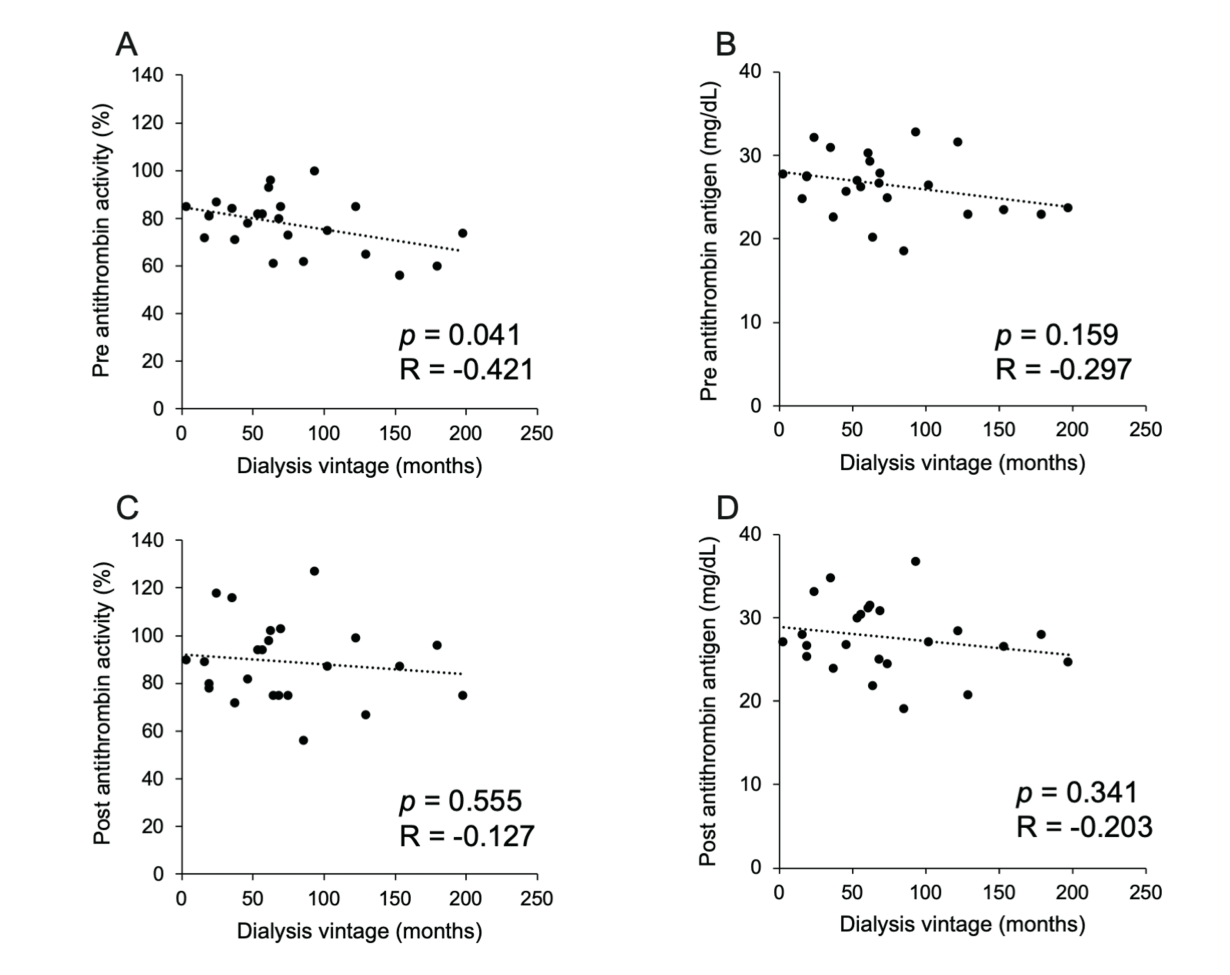
Relationship between AT activity, AT antigen levels before and after dialysis, and dialysis vintage. A: there is a significant positive correlation between before-dialysis AT activity and dialysis vintage. B: no correlation was found between before dialysis AT atigen and dialysis vintage. C and D: no correlation was found between after-dialysis AT activity or antigen levels and dialysis vintage. AT: antithrombin.

We investigated the correlation between pre-AT activity and antigen levels and dialysis vintage to analyze the effects of long-term heparin use on AT activity and antigen levels. The results showed a negative correlation between lower pre-AT activity and longer dialysis history (p = 0.041, R = -0.421, Figure 3A). However, there was no correlation between pre-AT antigen levels and dialysis vintage (Figure 3B). There was also no correlation between post-dialysis (post) AT activity or post-AT antigen levels and dialysis vintage (Figure 3C, 3D).

We also analyzed the correlation between pre-AT activity and antigen levels and the rate of body weight gain (body weight gain/DW) to determine the dilution effect of AT due to hypervolemia. No correlation was found in either case (p = 0.201, R = -0.271, and p = 0.355, R = -0.197, respectively).

Relationship between α1-MG and β2-MG reduction rates and AT or albumin leakage

**Figure 4:**
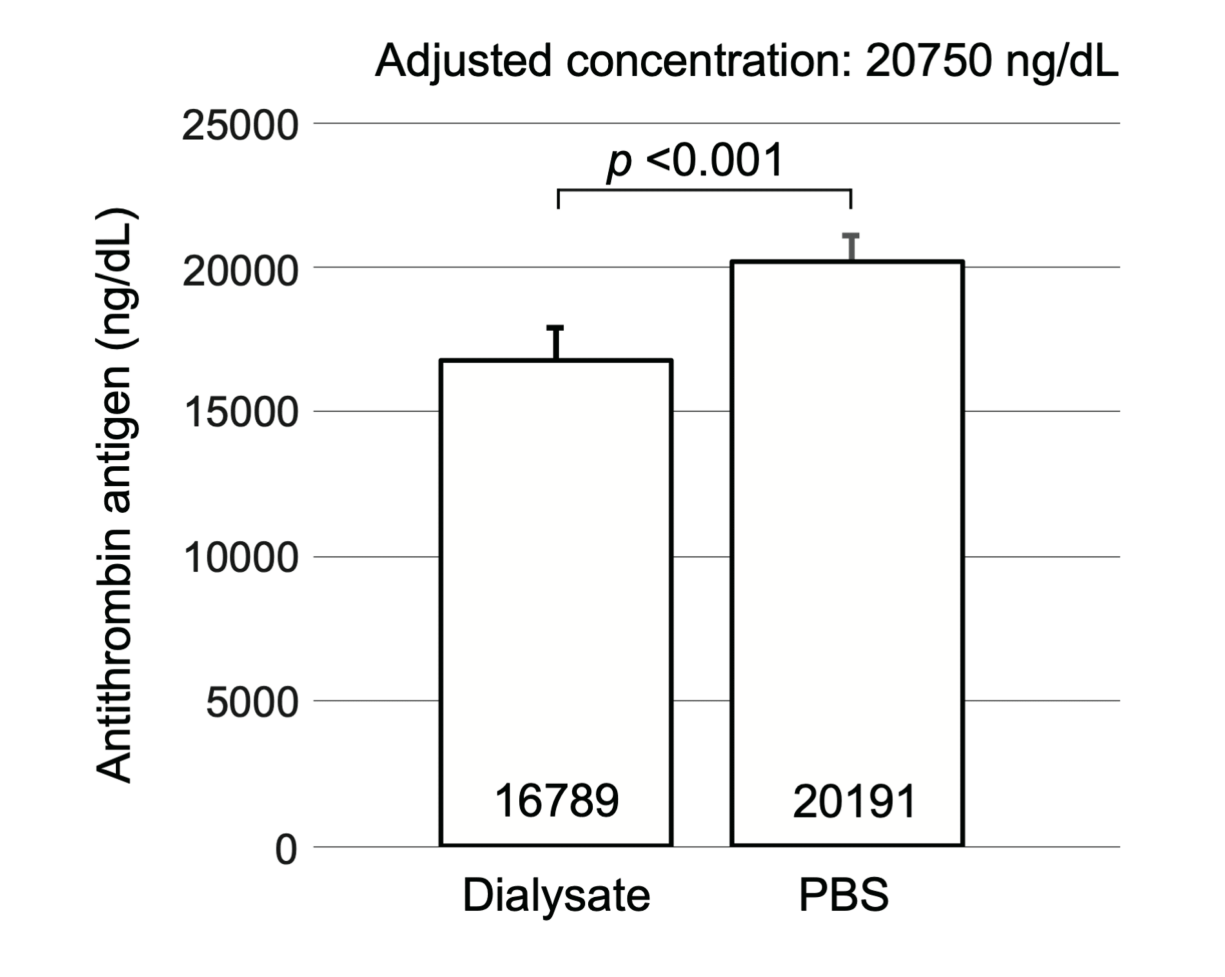
Comparison of dialysate and PBS when measuring AT concentration. AT antigen levels in the dialysate were 20% lower than AT antigen levels in PBS. AT: antithrombin; PBS: phosphate-buffered saline.

First, we measured purified AT antigen levels (adjusted to 20,750 ng/dL) diluted in the dialysate and PBS to confirm that AT antigen levels in the dialysate effluent could be accurately measured. The PBS dilution was measured similarly to the adjusted concentration, while the dialysate dilution was approximately 20% lower than that of the PBS dilution (Figure 4, p < 0.001).

AT leakage per dialysis session was 30.6 (6.0-53.1) mg/session in HDF therapy, significantly higher than in HD therapy (Table 3, p < 0.001). Among the hemodiafilters, the FIX-S series (n=8) showed the highest AT leakage, with 44.1 (IQR: 32.5-55.4) mg/session. Similarly, albumin leakage was 2166 (IQR: 443-4294) mg/session in the HDF therapy, which was significantly higher than that in the HD therapy (p < 0.001). However, no correlation was found between AT leakage and AT activity or antigen levels, either before or after dialysis.

**Table 3:** Antithrombin and albumin leakage from different dialysis mode. HD: hemodialysis; HDF: hemodiafiltration.

	All patients	Patients on HD	Patients on HDF	p-value for HD/HDF comparisions
Antithrombin leakage (mg/session)	2.4 (0.7-30.6)	0.7 (0.1-0.9)	30.6 (6.0-53.1)	<0.001
Albumin leakage (mg/session)	200 (108-2166)	108 (66-135)	2166 (443-4294)	<0.001

**Figure 5:**
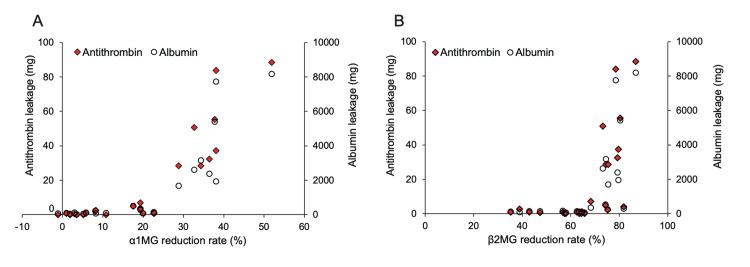
Relationship between albumin leakage, AT leakage, and α1-microglobulin and β2-microglobulin reduction rates. A: AT and albumin leakage increased rapidly when the a1-microglobulin reduction rate exceeded 25%. B: AT, and albumin leakage increased rapidly when the b2-microglobulin reduction rate exceeded 70%. AT: antithrombin.

Analysis of the relationship between a1-MG or b2-MG reduction rates and AT or albumin leakage showed that both AT and albumin leakages increased sharply when the a1-MG reduction rate exceeded 25% (Figure 5A) and the b2-MG reduction rate exceeded 70% (Figure 5B). Additionally, AT leakage patterns closely mirrored albumin leakage patterns.

Antithrombin adsorption on dialysis membranes under different dialysis mode

**Figure 6:**
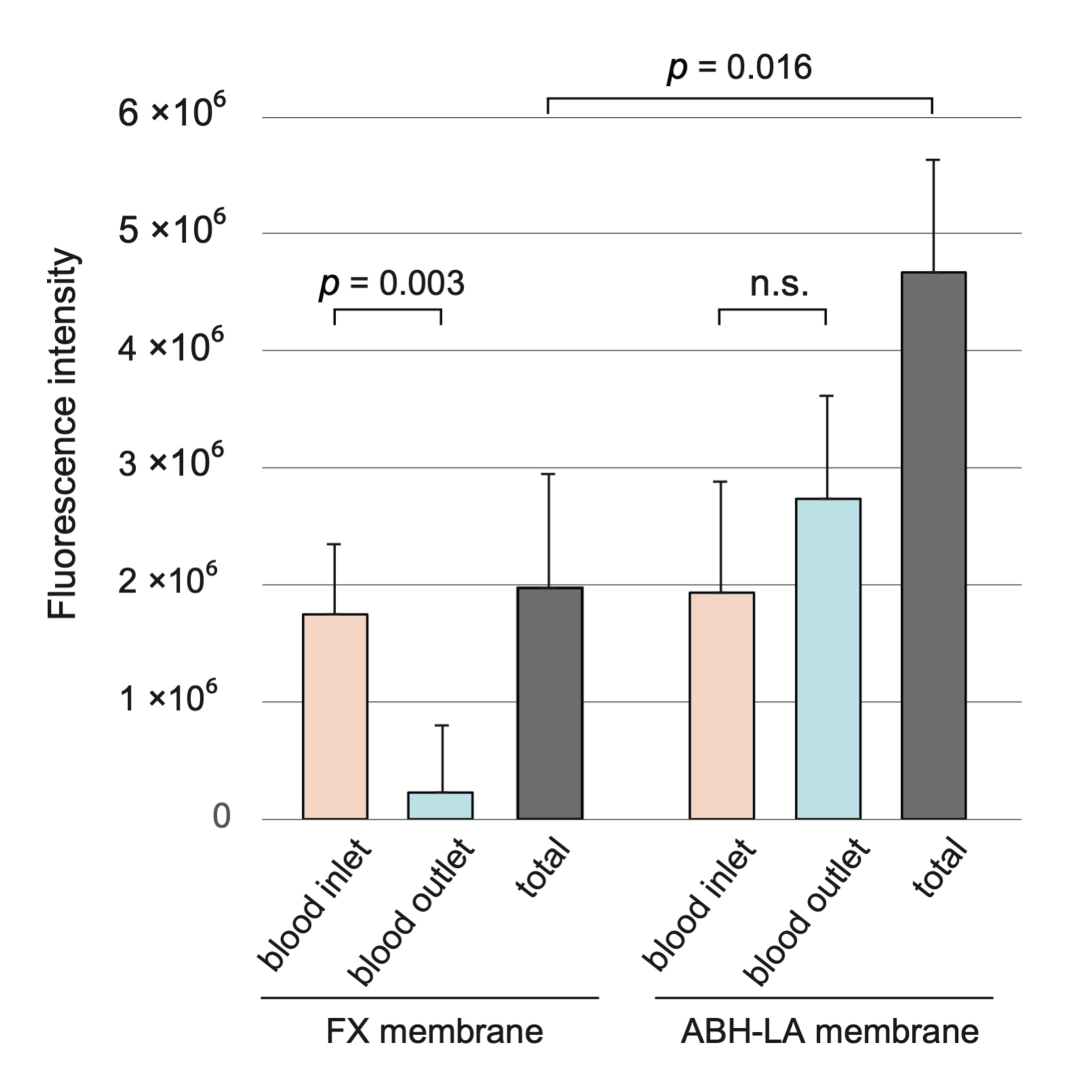
AT adsorption on FX and ABH-LA membranes. The amount of AT adsorbed on the FX and ABH-LA membranes is indicated by the fluorescence intensity. The total amount of AT adsorbed was significantly higher with the ABH-LA membranes (used in pre-diluted hemodiafiltration therapy) than with the FX membranes (used in hemodialysis therapy). For the FX membranes, the AT adsorption was higher on the blood inlet side than on the blood outlet side. For the ABH-LA membranes, no differences were observed between the blood inlet and outlet sides. AT: antithrombin.

Significant differences in AT leakage were observed depending on dialysis treatment type, such as HD and HDF therapy. Therefore, we also compared AT adsorption on the dialysis membrane across different conditions. We collected the dialysis membranes after therapy and performed immunofluorescence staining to semi-quantify the adsorbed AT proteins. When comparing the total adsorption on the blood inlet and blood outlet sides, the ABH-LA membrane (HDF therapy) showed significantly more AT adsorption (fluorescence intensity) than the FX membrane (HD therapy) (Figure 6, p = 0.016). Compared with the hollow fiber location, the FX membrane adsorbed significantly more AT protein on the blood inlet side than on the blood outlet side (p = 0.003). In contrast, no difference in AT protein adsorption was observed between the inlet and outlet of the ABH-LA membrane.

## Discussion

In this study, we observed reduced AT activity (45.8% of patients, below the reference range) and AT antigen levels (25.0% of patients, below the reference range) in patients undergoing HD. Given the low prevalence of inherited AT deficiency in the Japanese population is 0.15% [20], patients on HD were found to have a high frequency of acquired AT deficiencies. Our findings further suggest that long-term heparin use associated with dialysis therapy may contribute to decreased AT activity and antigen levels. Furthermore, we found that HDF therapy caused AT leakage, although this depended on the type of hemodiafilter used. However, no correlation was found between AT leakage and AT activity or antigen levels, indicating that AT leakage alone may not directly cause AT deficiency.

Pre-AT activity and antigen levels were significantly lower in patients on HD compared to healthy participants. Lai et al. [16] and Vaziri et al. [21] reported similar results. The primary cause of decreased AT activity is heparin use associated with dialysis therapy. Continuous heparin use, such as in heparin therapy, causes acquired AT deficiency, while intermittent use, such as in HD therapy, does not decrease AT activity [22]. However, in this study, a significant negative correlation was observed between pre-AT activity and dialysis vintage, which reflects a history of heparin use. Furthermore, SF and TAT, which indicate coagulation activation, and D-dimer, which indicates the presence of a thrombus, were elevated in patients on HD, suggesting that the coagulation system was activated by blood contact with the dialyzer and dialysis circuit during dialysis therapy. Therefore, AT may be reduced by the use of heparin in combination with dialysis therapy. This can be explained by the fact that the AT reduction rate in HD therapy, which has moderate removal efficiency, was significantly higher than the reduction rates of albumin and a1-MG. In summary, these results suggest that in dialysis therapy, even intermittent but long-term heparin use may decrease AT antigen levels owing to AT consumption, resulting in a corresponding decrease in AT activity.

To our knowledge, this is the first study to measure and analyze AT antigen levels in dialysate effluents. While albumin, α1-MG, β2-MG [23], BUN, and indole sulfate [24] have previously been measured in dialysate effluents, and comprehensive measurement of dialysate effluent using Liquid Chromatography Mass Spectrometry has also been reported [25]. However, no study has focused on AT hemodynamics in patients undergoing HD or measured AT antigen levels in dialysis effluents. Thus, we investigated the effect of AT protein leakage during dialysis therapy on the decrease in AT activity and antigen levels in patients undergoing HD. In our study, HD therapy resulted in minimal AT leakage, while HDF therapy resulted in a median leakage of 30.6 mg. The actual leakage may be higher, as AT levels measured in dialysate were approximately 20% lower than those in typical measurement conditions (diluted in PBS). Although no correlation was found between AT leakage and AT activity or antigen levels in patients on HD, AT protein leakage in HDF therapy may contribute to reduced AT levels over time.

We further examined AT adsorption onto dialysis membranes. A comparison of the total amount of adsorbed AT protein showed significantly higher adsorption on the ABH-LA membrane (HDF therapy) than on the FX membrane (HD therapy). Enhanced internal filtration [26] in modern dialysis membranes allows forward filtration on the blood inlet side, because the blood side has a higher pressure than the dialysate side, while back filtration occurs on the blood outlet side because the dialysate side has a higher pressure than the blood side [27]. Therefore, the FX membrane adsorbed more at the blood inlet side than at the blood outlet side. Additionally, in pre-diluted online HDF, stronger forward filtration throughout the hemodiafilter compared to HD may have resulted in greater adsorption on the ABH-LA membranes than on the FX membranes. These results suggest that under HDF conditions, both AT leakage and adsorption onto dialysis membranes increase, potentially impacting AT levels.

Decreased AT activity is a known risk factor for VA-related thrombosis [17,28]. While venous neointimal hyperplasia is the primary cause of VA-related thrombosis [29], acquired thrombophilia may play a role. Measuring AT activity may be beneficial in cases of frequent thrombosis of arteriovenous fistulas or grafts. Therefore, we recommend routine AT activity measurements to prevent thrombosis in patients undergoing HD. Furthermore, this study revealed that AT leakage can be predicted from the α1-MG and β2-MG reduction rates. In cases with decreased AT activity, a history of thrombotic shunt occlusion, or a thrombotic tendency, dialysis conditions such as HD therapy should be considered to prevent AT leakage by predicting AT leakage from α1-MG and β2-MG reduction rates.

This study had some limitations. First, the sample size was insufficient to robustly analyze the relationship between AT leakage and AT activity or antigen levels. Second, while immunofluorescence staining enabled a comparative analysis of AT protein adsorption between membranes, it did not provide quantification. 

## Conclusions

AT activity and antigen levels were significantly lower in patients on HD than in healthy participants. This decrease appears to result from long-term heparin use in dialysis therapy, combined with AT leakage and adsorption onto dialysis membranes. For patients with decreased AT activity or antigen levels, adjusting treatment conditions, such as adopting HD therapies to minimize AT leakage and adsorption onto the dialysis membrane is recommended. Furthermore, regular monitoring of AT activity is essential to manage the risk of thrombosis, including VA-related thrombosis. Additionally, research could investigate alternative anticoagulation strategies to minimize AT loss and assess their impact on hemodynamics.
